# Neurocognitive Therapeutic Exercise Integrated with Focal Mechanical Vibrations in a CANVAS Patient: A Case Report

**DOI:** 10.3390/neurolint18040070

**Published:** 2026-04-17

**Authors:** Filippo Camerota, Filippo Mario Topa, Giuseppe Di Pietro, Federico Zangrando, Lorenzo Coluccia, Massimiliano Mangone, Marco Paoloni, Andrea Truini, Claudia Celletti

**Affiliations:** 1 Physical Medicine and Rehabilitation Division, Umberto I University Hospital, 00185 Rome, Italy; filippo.camerota@uniroma1.it (F.C.); f.zangrando@hotmail.com (F.Z.); coluccia.2011171@studenti.uniroma1.it (L.C.); 2 Department of Anatomy, Histology, Forensic Medicine and Orthopedics, Sapienza University, 00185 Rome, Italy; filippomario.topa@uniroma1.it (F.M.T.); massimiliano.mangone@uniroma1.it (M.M.); marco.paoloni@uniroma1.it (M.P.); 3 Department of Human Neuroscience, Sapienza University, 00185 Rome, Italy; giuseppe.dipietro@uniroma1.it (G.D.P.); andrea.truini@uniroma1.it (A.T.); 4 Department of Life Sciences, Health, and Health Professions, Link Campus University, 00165 Rome, Italy

**Keywords:** CANVAS, focal mechanical vibration, gait analysis, stabilometry, neurocognitive rehabilitation, proprioception, vestibular areflexia

## Abstract

Cerebellar Ataxia, Neuropathy and Bilateral Vestibular Areflexia Syndrome (CANVAS) is a progressive multisystem disorder characterized by cerebellar ataxia, sensory neuropathy and bilateral vestibular failure. Although intensive rehabilitation is commonly recommended, the actual effectiveness and the most appropriate physiotherapeutic strategy for CANVAS have not been clearly established. Background/Objectives: To evaluate the effects of an integrated rehabilitation program combining neurocognitive therapeutic exercise and focal muscle vibration (FMV) on clinical and instrumental measures of gait, balance and postural stability in a CANVAS patient. Methods: A structured protocol consisting of neurocognitive therapeutic exercise and FMV was administered. Clinical measures included the Berg Balance Scale, Tinetti, SARA and SF-36. The instrumental evaluations included stabilometry and gait analysis. Results: The intervention produced improvements in balance scores associated with a reduction in fall risk. Stabilometry revealed reduction in oscillation area. Conclusions: FMV combined with neurocognitive therapeutic exercise may promote clinical and biomechanical improvements in CANVAS.

## 1. Introduction

Cerebellar Ataxia, Neuropathy and Bilateral Vestibular Areflexia Syndrome (CANVAS) is a late-onset, slowly progressive neurodegenerative disorder associated, in most cases, with biallelic intronic AAGGG repeat expansion in the RFC1 gene [1]. Although it is attracting increasing recognition, CANVAS remains largely underdiagnosed due to its heterogeneous presentation and the gradual evolution of the complete clinical triad [2]. Recent genetic studies suggest that the estimated prevalence of RFC1 CANVAS/spectrum disorder ranges from 1:20.000 to 1:625 [1].

Clinically, the ataxic syndrome is explained by a combination of cerebellar postural disorder, proprioceptive deficit due to sensory axonal neuropathy and/or vestibular deficit. The interaction of these components results in a complex multisensory integration deficit associated with postural instability and increased risk of falls [3]. Dysautonomia and chronic cough are frequently associated features and may precede neurological manifestations by several years [2]. Functional decline is largely driven by progressive balance impairment and locomotor instability, with particular impacts on gait, postural control, and activities of daily living.

Currently, no disease-modifying therapies are available for CANVAS. Management is supportive and mainly focused on fall prevention and vestibular rehabilitation [1]. In this setting, even if vestibular rehabilitation can promote gaze stabilization and central compensation [4], its effects on global postural control and gait may be limited, particularly in the presence of severe proprioceptive deficits related to sensory neuronopathy [1]. Therefore, rehabilitation strategies targeting proprioceptive dysfunction may represent a relevant therapeutic approach [5].

Focal mechanical vibration (FMV) is a proprioceptive neuromodulatory technique capable of selectively activating Ia afferents when delivered at frequencies around 100 Hz, inducing plastic changes at both spinal and cortical levels. Its integration in neurorehabilitation may enhance motor learning, sensorimotor integration, and postural control [6]. However, its application in CANVAS has not been previously described.

Neurocognitive rehabilitation is based on task-oriented exercises that require sensory discrimination and multisensory integration. These tasks engage kinesthetic information related to movement direction, amplitude, and spatial orientation, as well as tactile cues such as pressure and weight perception. By actively solving progressively structured motor problems, patients are encouraged to develop organizational strategies that may not be spontaneously available but can emerge through guided facilitation. This process may promote motor relearning and functional reorganization in pathological conditions [6].

Given the rarity of CANVAS, the absence of standardized rehabilitation protocols, and the limited evidence addressing proprioceptive-targeted interventions in this population, we report the case of a patient with genetically confirmed CANVAS treated with an integrated rehabilitation program combining FMV and neurocognitive therapeutic exercise. The objective was to evaluate the clinical and instrumental effects of this combined approach on postural stability and gait performance.

## 2. Case Presentation

A 59-year-old man presented with a 5-year history of progressively worsening gait instability, imbalance and frequent near-falls. The patient reported a persistent chronic cough over the past five years. Over time, symptoms progressively interfered with daily activities, particularly ambulation and balance-related tasks, leading to an increased risk of falls. The patient remained independently ambulant but reported reduced confidence while walking.

Laboratory investigations for acquired causes of polyneuropathy were negative, including screening for diabetes mellitus, renal, hepatic, and thyroid dysfunction, vitamin deficiencies, paraproteinemia, viral infections, and systemic inflammatory disorders. The patient had no history of neurological disease or exposure to neurotoxic agents.

Clinical examination revealed a broad-based ataxic gait with independent ambulation, absent deep tendon reflexes, a positive Romberg sign, impaired vibration sense at the hands and feet, bilateral vestibular areflexia, chronic cough, and signs of dysautonomia. A detailed nerve conduction study demonstrated a severe sensory neuropathy characterized by absent sensory action potentials at four limbs [7]. The diagnosis of CANVAS was confirmed by genetic testing revealing biallelic intronic repeat expansion in the RFC1 gene.

Following neurological evaluation, the patient was referred for rehabilitation, and a structured protocol was designed. The patient signed informed consent for the following experimental treatment program and for the publication of the clinical findings. This case report was prepared in accordance with the CARE reporting guidelines.

The rehabilitation program consisted of two sequential components: neurocognitive therapeutic exercise followed by FMV. The patient initially underwent 24 physiotherapy sessions (60 min each, twice weekly). Neurocognitive exercise was administered by a physiotherapist expert in this field of rehabilitation. The composition of the neurocognitive exercise included a perceptual hypothesis as a fundamental element. Faced with each problem, the patient had to develop a hypothesis regarding how it is possible to perceive certain differences within the task, and above all, how to recognize the differences that can be considered useful for solving the problem. The cognitive operations were divided into two categories: spatial, referring to spatial recognition operations using the body as a receptive surface (direction, distance, and orientation); and contact, referring to the modalities of interaction with the ground regarding the foot (touch, pressure, and weight).

Exercises were performed at three different levels: first-, second-, and third-degree. They were carried out progressively with increasing complexity for a total of 12 weeks of rehabilitation.

In first-degree exercises, the patient was asked to recognize, with his eyes closed, the characteristics of specific movements of segments of his body performed “passively” by the therapist. With second-degree exercises, the therapeutic work also included voluntary movements. Active contractions were performed, thus recruiting a certain number of motor units. Third-degree exercises recruit a greater number of motor units and involve performing gestures according to various spatial and temporal combinations to regulate the spatiality, temporality, and intensity of movement. A concrete example of a second-degree neurocognitive exercise involved the use of two scales, each placed under one foot of the patient while they were standing with their feet parallel. The patient was asked to transfer the load from right to left and vice versa in a programmed manner, with his eyes closed, while the perceptual task was to recognize the amount of load transferred. In a subsequent phase, the third-degree exercise involved the dual task of transferring the entire weight onto a single lower limb (contact task) and then performing programmed steps of different length or direction with the other limb (spatial task).

During the six first days of rehabilitation treatment the exercises were administered in the first- and second-degree modality for 30 min in supine position and for 30 min in standing position.

From the 7th to the 18th day, the exercise was in the second- and third-degree modality and was administrated in sitting position and standing position for half the total time, respectively.

The five last sessions were characterized by third-degree exercises executed in the standing position and in the walking training.

Subsequently, FMV was administered daily for six consecutive days. Focal muscle vibration was delivered using a specific device composed by an electromechanical transducer, a mechanical support, and an electronic control device (Cro^®^ System, NEMOCO srl, Roma, Italy). With the patient in the supine position, the transducer was applied perpendicular to the target muscle’s belly and vibration was delivered for 10 min to each of the following muscle groups bilaterally: quadriceps, tibialis anterior, hamstrings, triceps surae, and plantar fascia, for a total of 50 min per session. During the vibration therapy, a small contraction of the muscle being vibrated, lasting 15 s every two minutes, was requested; after the 10 min application, one minute of rest was observed. Vibration was applied to the distal part of the rectus femoris, the muscular belly of the tibialis anterior, and of the biceps femoris, in the muscle tendon junction of the triceps surae, and in the middle part of the plantar fascia for a total 50 min of therapy every day. The stimulation parameters of the vibration were constant at a frequency of 100 Hz and an amplitude of 0.1–0.5 mm as previously described.

The intervention was well tolerated, and no adverse events or undesirable symptoms were reported during the treatment period. Clinical and instrumental assessments were performed at five time points: before rehabilitation (T0); immediately after completion of neurocognitive therapy and prior to FMV (T1, clinical scales only); immediately after the FMV cycle (T2); 40 days after FMV treatment (T3, clinical scales only); and at 3-month follow-up (T4). Outcome measures included validated clinical scales as well as instrumental gait and postural analyses, as summarized in Table 1.

Balance and functional outcomes were assessed using:

- The Berg Balance Scale (BBS). The BBS was used to assess balance ability and fall risk in older adults. It consists of 14 items, each rated on a 5-point scale (0–4), with a maximum score of 56 [8]. The Minimal Clinically Important Difference (MCID) for improvement in balance as measured by the BBS varies from 3 points in multiple sclerosis [9] to 11.5 points in older adults with hip fractures [10].
- Tinetti Scale: is a validated clinical instrument for evaluating balance and gait in older adults. The assessment encompasses measures of static, dynamic, reactive, and anticipatory balance, as well as ambulation and transfer capabilities, providing a comprehensive overview of an individual’s mobility status. The scores range from 1 to 28, where a score of 28 indicates a low risk of falling [11]. The MCID for the Tinetti Performance-Oriented Mobility Assessment (POMA) total score is generally recognized as a change of 3 to 4 points. Also, MCID can vary based on the specific clinical population [12].
- SARA (Scale for the Assessment and Rating of Ataxia) is a tool for assessing ataxia. It has eight categories with an accumulative score ranging from 0 (no ataxia) to 40 (most severe ataxia). The scale is made up of 8 items related to gait, stance, sitting, speech, finger-chase test, nose-finger test, fast alternating movements and heel-shin test [13]. Intra-rater and inter-rater reliability were found to be high for SARA when it was used to assess ataxia [14].

An MCID of 1.5 points has been reported for SARA. [15].

- SF-36. Short Form-36 is a questionnaire for evaluating quality of life (SF-36). The SF-36 measures eight scales: physical functioning (PF), role physical (RP), bodily pain (BP), general health (GH), vitality (VT), social functioning (SF), role emotional (RE), and mental health (MH) [16]. SF-36 was administered only at T0, T2 and T4 time points.

Instrumental evaluations included stabilometry and gait analysis. Postural stabilometry with the oscillatory area sway and center of pressure (CoP) length parameters was executed: the subject was instructed to maintain an upright standing position for 30 s with arms along his sides and feet positioned over sketches representing the foot with an angle of 30° with respect to the anteroposterior (AP) direction. The participant was asked to maintain an upright standing position with eyes open (OE), looking straight ahead for the first trial, and was then requested to keep his eyes closed (CE). Three trials were performed, and mean values were used for analysis. The parameters obtained were the length (mm) and the area (mm^2^) described by the center of pressure. Postural stabilometry was performed using the BTS Infinity baropodometric platform. Data were sampled at 40 Hz. The test–retest reliability of CoP-based stabilometric parameters in quiet stance conditions has been established in the literature, with intraclass correlation coefficients (ICCs) generally ranging from 0.80 to 0.95 for CoP path length and sway area [17].

The gait analysis evaluation was performed by a skilled rehabilitation doctor at T0, T2, and T4 and was comprehensive, including kinematic, kinetic, and surface EMG assessments. Gait analysis was conducted using a 3D optoelectronic system (Smart D500; BTS Bioengineering, sampling frequency 340 Hz), a force platform (Kistler, sampling frequency of 1000 Hz), two TV camera video systems (BTS Bioengineering), and up to 16 sEMG probes (FreeEMG, BTS Bioengineering, sampling frequency 1000 Hz).

In accordance with the European Recommendations for surface Electromyography [18] and the Atlas of Muscle Innervation Zones [19] we acquired 10 muscles: gluteus medius (GM), rectus femoris (RF), semitendinosus (ST) tibialis anterior (TA), gastrocnemius medialis (GasM). Surface EMG electrode placement was performed according to SENIAM recommendations [18]. Prior to electrode application, the skin was prepared by light abrasion and cleansing with isopropyl alcohol to reduce impedance and ensure signal quality. All surface EMG electrode placements across all assessment time points were performed by the same trained operator to minimize intra-rater variability. The intra-rater reliability of sEMG placement using SENIAM guidelines in gait analysis settings has been reported to be good to excellent (ICC > 0.85) [20].

Markers were positioned on the participant’s body, as described by Davis et al. [21]. Spatiotemporal parameters, including step length, cadence, walking speed, and phase durations, were recorded and automatically computed by the BTS Smart Analyzer software. The gait cycle was defined as the interval between two successive ipsilateral heel strikes, identified from ground reaction force data acquired via the Kistler force platform. Heel strike and toe-off events were detected using standard force threshold criteria, as previously described in standard clinical gait analysis protocols [21]. The test retest reliability of spatiotemporal gait parameters obtained with 3D optoelectronic systems has been demonstrated to be good to excellent, with ICC values typically exceeding 0.85 for walking speed, cadence, and step length [22].

### Results

The chronological sequence of clinical events and rehabilitation phases is summarized in Table 1. The results of the clinical scales are shown in Table 2. Table 3 contains a summary of the main results of the postural and gait analysis evaluation, which include significant results. 

The BBS and Tinetti scores showed an improvement in parallel while SARA decreased, and these data are also associated with an instrumental improvement of in stability (e.g., a reduction in CoP sway area).

The patient reported a clear subjective improvement in balance and walking confidence following the rehabilitation program, particularly after the vibration phase. He experienced greater stability during daily activities and reduced fear of falling.

## 3. Discussion

CANVAS is a rare, progressive neurodegenerative disorder that may clinically overlap with other adult-onset ataxias, including spinocerebellar ataxias, Friedreich’s ataxia, and multiple-system atrophy of the cerebellar type. The diagnosis is frequently delayed because early manifestations—such as chronic cough or isolated sensory impairment—may precede the complete clinical triad by several years. In the present case, progressive gait instability evolved over five years before genetic confirmation was obtained, reflecting the typical gradual course of the disease [1].

The differential diagnosis was carefully considered. The presence of severe sensory neuronopathy with absent sensory action potentials in all four limbs, in the absence of motor nerve involvement on nerve conduction studies, strongly supported CANVAS, as this pattern is uncommon in most spinocerebellar ataxias. The absence of extrapyramidal features, pyramidal signs, and significant comorbidities such as cardiomyopathy or diabetes further reduced the likelihood of alternative diagnoses, including Friedreich’s ataxia and MSA-C [1]. Definitive diagnosis was established by identification of biallelic intronic repeat expansion in the RFC1 gene.

Current management of CANVAS is supportive. Vestibular rehabilitation has been shown to improve gaze stabilization and functional outcomes [23,24]. However, gait and postural instability in CANVAS reflect the combined involvement of cerebellar degeneration and severe proprioceptive impairment due to sensory neuronopathy. The gait alterations described by Di Rauso et al. [25], including reduced ankle dorsiflexion and abnormal plantar flexor activity, further suggest that peripheral mechanisms contribute substantially to instability.

In this context, the present case supports the rationale for integrating proprioceptive-targeted interventions into rehabilitation programs. The patient showed clinically meaningful improvements in balance and fall risk following neurocognitive therapeutic exercise, with further enhancement after FMV. Improvements were observed across balance scales showing a global increase in all the score that were higher than the values indicated as MCID in the literature and were maintained at follow-up. Ataxia severity based on SARA decreased in parallel with balance improvement, suggesting a functional interaction between postural control and cerebellar performance.

Postural stabilometry demonstrated differential behavior under open- and closed-eye conditions, supporting the hypothesis that proprioceptive modulation contributed to improved sensory integration. This hypothesis is related to the marked reduction in the sway area in the closed-eye test after FMV, where the proprioceptive role is most evident. A similar mechanism could explain the reduction in cadence indicating greater stability.

The neurophysiological rationale is consistent with evidence showing that FMV selectively activates Ia afferents and may induce adaptive plasticity at spinal and cortical levels [26]. Combined with neurocognitive exercise aimed at enhancing multisensory integration, this approach may facilitate compensatory mechanisms in a multisystem disorder such as CANVAS. Although it is limited to a single case and does not allow causal inference, this report highlights the potential role of proprioceptive-oriented rehabilitation strategies in a condition where sensory neuronopathy is a central pathophysiological feature.

This report has inherent limitations due to its single-case design, which does not allow causal inference regarding the specific contribution of focal mechanical vibration. Although improvements in balance and fall risk were observed, the sequential administration of rehabilitation and vibration does not permit clear attribution of effects to a single intervention. Alternative explanations, including natural symptom fluctuation, learning effects from repeated testing, placebo or attention-related influences, and regression to the mean, cannot be excluded. Larger controlled studies are needed to clarify the potential additive or synergistic effects of combined neurocognitive rehabilitation and FMV.

The sequential administration of neurocognitive rehabilitation followed by FMV was intended to consolidate motor strategies before introducing proprioceptive stimulation; however, this design does not allow clear separation of the individual effects of each intervention. Moreover, the absence of consistent changes in global gait parameters suggests that the observed modifications in cadence and step width should be interpreted cautiously, as they may reflect adaptive strategies rather than structural biomechanical changes.

## 4. Conclusions

Association between FMV therapy and neurocognitive therapeutic exercise may clinically and instrumentally improve balance in CANVAS. Within the significant limitation inherent in case reports, our results suggest that integrating proprioceptive vibration within neurorehabilitation protocols for complex ataxic disorders may be useful. We encourage further research incorporating control and CANVAS individuals.

## Figures and Tables

**Table 1:** Chronological timeline of clinical course and rehabilitation program.

Time Point	Clinical Events
About 5 years before rehabilitation	Onset of progressive gait instability, imbalance, and chronic cough
4–5 years before rehabilitation	Gradual development of sensory impairment and bilateral vestibular areflexia
Diagnostic phase	Neurological evaluation, nerve conduction studies showing severe sensory neuropathy; genetic confirmation of RFC1 biallelic expansion
T0	Baseline clinical and instrumental assessment
Weeks 1–12	24 sessions of neurocognitive therapeutic exercise (twice weekly)
T1	Post-exercise evaluation (prior to FMV)
Week 13	FMV cycle (6 consecutive days)
T2	Immediate post-FMV
+40 days (T3)	Follow-up evaluation
+3 months (T4)	Final follow-up evaluation

**Table 2:** Results of the scale administered to the patient at different times.

	T0	T1	T2	T3	T4	Variation from Baseline
Tinetti score for balance (max score 16)	10	14	16	15	14	
Tinetti score for gait (max score 12)	6	10	12	12	12	
Tinetti Score (max 28)	16	24	28	27	26	+12 (T2) +10 (T4)
Berg Balance Scale (max score 56)	43	52	56	56	55	+13 (T2) +12 (T4)
SARA (0–40)	7	4	1	1	2	−6 (T2) −5 (T4)
SF-36 PF	50	-	65	-	60	
SF-36 RP	25	-	25	-	75	
SF-36 RE	33.3	-	0	-	100	
SF-36 VT	25	-	25	-	45	
SF-36 MH	20	-	36	-	28	
SF-36 SF	12.5	-	37.5	-	0	
SF-36 BP	45	-	67.5	-	45	
SF-36 GH	35	-	55	-	50	

**Table 3:** Results of the postural evaluation and gait analysis examination.

	T0	T2	T4
Stabilometry			
Sway area OE (mm^2^)	6949	10,379	3345
Sway area CE (mm^2^)	30,340	30,905	26,224
Gait analysis evaluation			
Velocity (m/s)	0.99 ± 0.00	0.99 ± 0.03	0.96 ± 0.07
Mean velocity (%/s)	55.49	55.05	53.33
Cadence	108.6	99.6	95.8
Step width (m)	0.08	0.07	0.09

## Data Availability

The data presented in this case report are available from the corresponding author upon reasonable request.

## Abbreviations

The following abbreviations are used in this manuscript:

FMV	Focal Muscle Vibration
SARA	Scale for the Assessment and Rating of Ataxia
CANVAS	Cerebellar Ataxia, Neuropathy and Bilateral Vestibular Areflexia Syndrome
BBS	Berg Balance Scale
SF-36	Short Form -36
